# The Replacement of 10 Non-Conserved Residues in the Core Protein of JFH-1 Hepatitis C Virus Improves Its Assembly and Secretion

**DOI:** 10.1371/journal.pone.0137182

**Published:** 2015-09-04

**Authors:** Loïc Etienne, Emmanuelle Blanchard, Audrey Boyer, Virginie Desvignes, Julien Gaillard, Jean-Christophe Meunier, Philippe Roingeard, Christophe Hourioux

**Affiliations:** 1 Inserm U966, Faculté de medicine, Université François Rabelais and CHRU de Tours, Tours, France; 2 Plate-forme des Microscopies, PPF ASB, Université François Rabelais and CHRU de Tours, Tours, France; Saint Louis University, UNITED STATES

## Abstract

Hepatitis C virus (HCV) assembly is still poorly understood. It is thought that trafficking of the HCV core protein to the lipid droplet (LD) surface is essential for its multimerization and association with newly synthesized HCV RNA to form the viral nucleocapsid. We carried out a mapping analysis of several complete HCV genomes of all genotypes, and found that the genotype 2 JFH-1 core protein contained 10 residues different from those of other genotypes. The replacement of these 10 residues of the JFH-1 strain sequence with the most conserved residues deduced from sequence alignments greatly increased virus production. Confocal microscopy of the modified JFH-1 strain in cell culture showed that the mutated JFH-1 core protein, C10M, was present mostly at the endoplasmic reticulum (ER) membrane, but not at the surface of the LDs, even though its trafficking to these organelles was possible. The non-structural 5A protein of HCV was also redirected to ER membranes and colocalized with the C10M core protein. Using a Semliki forest virus vector to overproduce core protein, we demonstrated that the C10M core protein was able to form HCV-like particles, unlike the native JFH-1 core protein. Thus, the substitution of a few selected residues in the JFH-1 core protein modified the subcellular distribution and assembly properties of the protein. These findings suggest that the early steps of HCV assembly occur at the ER membrane rather than at the LD surface. The C10M-JFH-1 strain will be a valuable tool for further studies of HCV morphogenesis.

## Introduction

The WHO has estimated that about 150 million people worldwide are chronically infected by hepatitis C virus (HCV) and are, thus, at risk of developing cirrhosis and liver cancer. HCV is an enveloped virus from the genus *Hepacivirus*, which belongs to the Flaviviridae family [1]. It has a single-stranded positive-sense RNA genome of ~ 9.6 kb encoding a single open reading frame (ORF) flanked, on both sides, by untranslated regions. The ORF encodes a precursor polyprotein of about 3,000 amino-acids, which is processed by cellular and viral proteases to produce three structural proteins (the core protein and the E1 and E2 envelope proteins), the viroporin p7, and six non-structural (NS) proteins (NS2, NS3, NS4A, NS4B, NS5A and NS5B). Since 2005, the JFH-1 strain, a genotype 2 virus, has been used to generate infectious HCV in cell culture (HCVcc) [2], making it possible to study the essential steps of the viral life cycle, including virus assembly and release, *in vitro* [3,4]. These late stages of the viral cycle are thought to take place at an interface between the surface of the cytoplasmic lipid droplets (LDs) and the cytosolic side of the endoplasmic reticulum (ER) membranes [5–8]. All three structural proteins are involved in the formation of viral particles, but the core protein is the basic building block for the nucleocapsid and this protein drives viral budding [9]. Many other functions have been proposed for this protein [10], but its sequence and structure suggest that it plays key roles in viral RNA encapsidation and in the acquisition of membrane-anchored envelope glycoproteins during HCV budding [4,11]. This protein is 191 amino-acids long and has three distinct domains. Domain 1 (D1), which contains highly basic residues and corresponds to approximately the first 120 amino-acids, is involved in RNA binding and protein homodimerization [12,13]. Domain 2 (D2), which is located between amino-acids ~ 120 and 180 consists of a hydrophobic domain structured into two amphipathic α-helices connected by a hydrophobic loop [14], and is responsible for the association of core with LDs and ER membranes [14,15]. Domain D3, corresponding to the C-terminal 10–12 amino-acids, contains a signal sequence involved in targeting the E1 envelope glycoprotein to the ER membrane. Cleavage of this domain by the signal peptide peptidase (SPP) leads to the release of the mature form of the core protein (about 180 amino-acids long), which is then trafficked towards the LD surface [16]. HCV can be efficiently propagated in naïve Huh7 hepatoma cells, but it is not currently possible to observe HCV assembly and morphogenesis in this model. The titers of infectious viral particles generated in the HCVcc system suggest that there are probably too few assembly events per infected cell for detection by electron microscopy (EM) [4]. The site of viral budding has yet to be clearly identified, but the targeting of the core to the ER, together with the E1 and E2 envelope glycoproteins [17–19], and the dependence of HCV envelopment on VLDL synthesis suggest that this process probably occurs at the ER membranes, leading to the release of infectious particles from the host cell via the secretory pathway [20–22]. Viral RNA replication is independent of the structural proteins, but the assembly and release of infectious particles require almost all the viral proteins [3,23,24]. Studies with the HCVcc system have shown that the early phases of HCV assembly involve a complex and coordinated process, including the interaction of the core protein with LDs and the recruitment of other viral components by these organelles, to facilitate the initial steps of virus assembly [25]. It has therefore been suggested that core trafficking to LDs is an essential step in the production of infectious viruses, following cleavage of the D3 domain by the SPP to generate the mature core protein [26,27]. Indeed, current models describing the early phase of virion assembly on or near the LD surface were developed from observations that mutations affecting residues in the D2 domain and abolishing the trafficking of the core protein to LDs, severely hampered virus assembly [6,28]. In addition, the interaction between the core and NS5A proteins, accounting for the recruitment of NS5A to the LD surface, suggests that the delivery of viral RNA to the nascent HCV nucleocapsids is dependent on LDs [29–31]. However, the mechanisms by which nucleocapsids acquire their envelope remains largely unknown. It is currently thought that a tight interface is formed between the ER and core-coated LDs, allowing the nucleocapsids to bud and to be enveloped in ER membranes enriched in the NS2 protein, which could act as a platform bringing together the E1 and E2, p7, NS3 and NS5A proteins [32,33]. Recent studies of chimeric HCV genomes bearing the NS protein sequences of JFH-1 and structural protein sequences from another genotype 2 strain (J6 in the Jc1 chimera) or another strain of different genotype have suggested that core accumulation on LDs is inversely correlated with the efficiency of virus production [31,34–36]. This suggests that the JFH-1 core protein may be less efficient for viral assembly and/or secretion than the J6 core protein present in the genotype 2 Jc1 chimera or other HCV core proteins present in various intergenotypic chimeras.

We hypothesized that the JFH-1 core protein might have specific sequence features accounting for the lower titers observed in the HCVcc system for this particular strain than for various chimeras. We compared more than 1000 HCV core sequences, with the aim of identifying consensus/conserved regions. We identified 10 residues in the JFH-1 core protein sequence that were particularly unusual with respect to the other sequences considered. The replacement of these residues with more conserved amino-acids greatly increased virus production in the HCVcc model, demonstrating that the defective assembly and secretion observed with the JFH-1 strain were mostly due to the native core sequence of this strain. Consequently, the viral titers obtained with the JFH-1 strain can easily be increased by selective mutations of its sequence.

## Materials and Methods

### Sequence analysis

All HCV sequences were obtained from the HCV database of Los Alamos (http://hcv.lanl.gov), using the “alignment” tool provided in the “background information” menu. We chose to obtain all the complete HCV genomes available, to constitute a potentially more biological relevant source of functional circulating viruses for the *in silico* translation of viral sequences into HCV polyproteins. The sequences were first imported into an *ad hoc* database managed with Filemaker 12 software. Data curating, *in silico* translation, and sequence comparisons were accomplished with dedicated custom-developed Filemaker scripts, together with bioinformatics softwares (Bioedit, Clustalw), which were used to address more specific questions. Protein sequences were aligned and the consensus sequences identified were first compared with three reference sequences, corresponding to the genotype 1a Dj core (GenBank accession number AF529293, previously described in [37]), and the two genotype 2 core sequences extracted from the JFH-1 clone [2] and the J6CF strain [38].

### Plasmids

The Semliki forest virus (SFV) expression vector (pSFV1, Invitrogen) was used as a vector for subcloning of the JFH-1 and J6CF core sequences, according to the same strategy described for the Dj core sequence [7]. Various JFH-1 core mutants were generated with overlapping mutagenesis PCR based on the wt-JFH-1 core sequence as template and also cloned in the pSFV1 vector. Briefly, core nucleotide sequences were re-amplified with primers carrying *Bam*H1 sites at both their ends and inserted into the unique *Bam*H1 site present in pSFV1. The resulting constructs were checked by sequencing, linearized with *Spe*I and transcribed *in vitro* from the SP6 RNA promoter located upstream from the 5’ SFV UTR. We then electroporated BHK-21 and FLC4 cells with the RNA produced. As a control, we synthesized recombinant RNA encoding β-galactosidase (β-gal), with the pSFV3 (Invitrogen) expression vector. HCVcc experiments were conducted with plasmids pJFH-1 and pJFH-1/GND (kindly provided by Dr Wakita, National Institute of Infectious Diseases, Tokyo, Japan) [2]. C10M-JFH-1 was obtained by replacing the JFH-1 core sequence with the corresponding C10M mutant sequence by standard PCR-based cloning techniques. Detailed cloning information is available on request. The pFL-J6/JFH plasmid (kindly provided by Dr Rice, The Rockefeller University, New York, USA) corresponds to a chimeric genotype 2a HCV cDNA clone reconstructed by the insertion of the J6CF core-NS2 region into the JFH-1 genome [39]. All these plasmids carrying HCV genomes were linearized by digestion with *Xba*I and treated with mung bean nuclease (New England Biolabs) to remove four nucleotides to obtain the correct 3’ end of the HCV sequence. The digested plasmids were purified and used as a template for RNA synthesis. HCV RNA was synthesized *in vitro* with a T7 RiboMAX Express Large-Scale RNA kit (Promega). After DNase I treatment, RNAs were purified and integrity was checked by gel electrophoresis. Before electroporation of Huh7.5 cells, RNAs were quantified by spectrophotometry to normalize transfections.

### Cell culture and RNA transfection

BHK-21 cells (purchased from the ATCC) were maintained at 37°C in Glasgow minimal essential medium supplemented with 5% fetal calf serum and 8% tryptose phosphate. The human hepatocellular carcinoma cell line FLC4 [40] (a gift from Dr Yoshiharu Matsuura, Research Institute for Microbial Diseases, Osaka, Japan) was maintained in Dulbecco’s modified Eagle’s medium supplemented with 10% fetal calf serum. BHK-21 or FLC4 cells were electroporated with recombinant SFV RNA encoding the various HCV core proteins, as previously described [37]. For transfection, we mixed 8 x 10^6^ cells with 5 μg of recombinant SFV RNA and electroporated them by a single pulse at 350 V, 750 μF (Gene Pulser Xcell™ Eukaryotic System, Biorad). The electroporated cells were then diluted in growth medium, plated in 75 cm^2^ culture dishes, and cultured for 16 h at 37°C before treatment with trypsin for subsequent analysis. For confocal microscopy, transfected cells were cultured directly on glass coverslips in 24-well plates, at a density of 2 x 10^4^ cells per coverslip. The Huh7-derived cell clone Huh7.5 [41] (kindly provided by Dr Rice, The Rockefeller University, New York, USA), which is highly permissive for HCV RNA replication, was used for transfection and infection assays. 10^7^ cells were transfected with 20 μg of the different HCV RNAs and thereafter were grown in 10 cm dishes in Dulbecco’s modified Eagle’s medium supplemented with 100 units of penicillin per mL, 100 μg of streptomycin per mL and 10% fetal bovine serum. 24h post transfection cells were extensively washed with fresh culture medium to remove *in vitro* transcribed viral RNAs and allowed the cells to grow one additional day to exclude probability of quantification of input HCV RNAs. Electroporation with RNA was carried out with the same settings used for the BHK-21 and FLC4 cell lines.

### Electron microscopy and immunogold labeling

Transfected cells were fixed by incubation for 48 h in 4% paraformaldehyde and 1% glutaraldehyde in 0.1 M phosphate buffer (pH 7.2). They were then post-fixed by incubation for 1 h with 1% osmium tetroxide. They were dehydrated in a graded series of acetone solutions and the final cell pellets were embedded in Epon resin, which was allowed to polymerize for 24 h at 60°C. Ultrathin sections were cut, stained with 1% uranyl acetate and 1% lead citrate, placed on electron microscopy (EM) grids coated with collodion membrane, and observed with a Jeol 1011 electron microscope (Tokyo, Japan).

For immunogold labeling, ultrathin sections were treated for 10 minutes with 10% hydrogen peroxide, to dissolve the resin polymer. The grids were washed several times in PBS, and incubated with anti-HCV C7–50 monoclonal antibody (Abcam) diluted 1:50 in PBS supplemented with 1% BSA for 90 minutes at room temperature. Grids were washed several times in PBS and then incubated for 90 minutes at room temperature with the secondary anti-mouse antibody conjugated to gold particles (15 nm in diameter, British Biocell International, Cardiff, UK), diluted 1:40 in PBS. Sections were washed in PBS, fixed in 4% glutaraldehyde in PBS and stained as described above.

### HCV protein immunostaining, lipid droplet staining and confocal microscopy imaging

Cells grown on glass coverslips were washed with PBS and fixed by incubation for 30 minutes at room temperature in 4% paraformaldehyde in PBS. The reaction was blocked by incubation for 10 minutes at room temperature with 100 mM glycine in PBS, and cells were permeabilized by incubation for 30 minutes in 0.05% saponin, 0.2% bovine serum albumin (BSA) in PBS. Cells were then incubated for 30 minutes with anti-HCV core antibody diluted 1:200 in permeabilization buffer, in a dark, humid chamber. They were then washed in PBS for 15 minutes and incubated with the corresponding secondary antibody coupled to Alexa Fluor 488 (Molecular Probes, Life Technologies, OR), diluted 1:1000 in permeabilization buffer. Core protein was detected together with NS5A and the envelope E2 protein, according to the same protocol, with sheep polyclonal anti-NS5A antibody (kindly provided by Dr Harris, University of Leed, UK) diluted 1/200 and the human monoclonal anti-E2 AR3A antibody (kindly provided by Dr Law, SCRIPPS, CA, USA) diluted 1/200, respectively, followed by secondary antibodies coupled to Alexa Fluor 594. For lipid staining, cells were treated with Nile red (Sigma Aldrich), diluted 1:1000 (from a 1 mg mL^-1^ stock solution in acetone) in permeabilization buffer, during incubation with the secondary antibody. Cells were washed and mounted in 25 mM Tris pH 8.8, 5% glycerol, 2.5% 1,4-diazabicyclo [2,2,2] octane, 10% poly(vinyl alcohol) with a MW range of 31,000–50,000 (Sigma Aldrich). Indirect immunofluorescence and lipid droplet staining were analyzed under an Olympus Fluoview 500 confocal laser-scanning microscope (Olympus, Japan). The extent of colocalization of the two labels was measured with the “Colocalization” module of Imaris 7.2.3 X 64 (Bitplane AG, Saint Paul, MN). The extent of colocalization was assessed by determining the Pearson’s coefficient. The Pearson coefficient measures the correlation between the intensities of the two labels displaying colocalization in stacks of confocal sections acquired in two channels. This method provides a statistical evaluation of protein colocalization. Pearson’s coefficient is a number between +1 and-1, with positive values indicating a direct correlation, negative values indicating an inverse correlation, and values near 0 indicating no correlation.

### Titration of infectious HCV

Cell supernatants were subjected to serial 10-fold dilution in complete DMEM and were used to infect 10^4^ naïve Huh7.5 cells per well, in 96-well plates. All assays were performed in triplicate. The level of HCV infection was determined 3 days post-infection, by immunofluorescence staining for HCV core. The viral titer is expressed as focus-forming units per milliliter of supernatant (FFU/mL), calculated as the mean number of core-positive foci detected at the highest dilutions. For intracellular infectivity assays, infected cells were washed with PBS, and lysed by incubation in 1 mL of water to induce hypo-osmotic shock. Lysates were clarified by centrifugation at 10,000 x *g* for 10 minutes at 4°C, and used to inoculate naïve Huh7.5 cells, as for the determination of extracellular infectivity.

### Quantification of HCV RNA

Infected cells were lysed and total cellular RNA was isolated with a Nucleospin RNA kit (Macherey-Nagel), according to the manufacturer’s instructions. Initial cDNA synthesis was performed with 200 ng RNA, in a final volume of 20 μL, with random hexamers and the Superscript™ III Platinum® one-step quantitative RT-PCR system (Invitrogen). Quantitative PCR was carried out with two pairs of nucleotide primers (sequences presented in 5’ to 3’ form), targeting the 5’NC of HCV genome (TCTGCGGAACCGGTGAGTA / TCAGGCAGTACCACAAGGC), and the actin transcript (CGCACCACTGGCATTGTCAT / TTCTCCTTGATGTCACGCAC) used as reference, respectively. HCV transcript levels were determined relative to a standard curve generated for serial dilutions of a plasmid containing the HCV JFH-1 cDNA. Reactions were run in triplicate with SYBR green I master mix (Roche Applied Sciences) and a Roche LightCycler 480. HCV RNA levels in infected cell supernatants were quantified with the Abbott RealTime HCV assay, on an Abbott m2000_sp_-m2000_rt_ platform.

### HCV core quantification

HCV core from both infected cell lysates and supernatants was quantified in a fully automated microparticle chemiluminescence immunoassay (Architect HCV Ag; Abbott). Briefly, infected cells were harvested by trypsin treatment and washed in PBS. The cells were centrifuged, and the resulting pellet was treated with a lysis buffer (1 M Tris pH 8, 1 mM EDTA, 1% NP40) supplemented with a protein inhibitor cocktail (1 mM phenylmethylsulfonyl fluoride, 2 mg mL^-1^ aprotinin, 2 mg mL^-1^ leupeptin). The resulting cell lysates were clarified by centrifugation and serially diluted for core quantification. For core titration in supernatants, a brief centrifugation (1500 x *g* / 5 minutes) was initially performed to remove cell debris.

## Results

### Sequence analysis and mutagenesis

**Fig 1:**
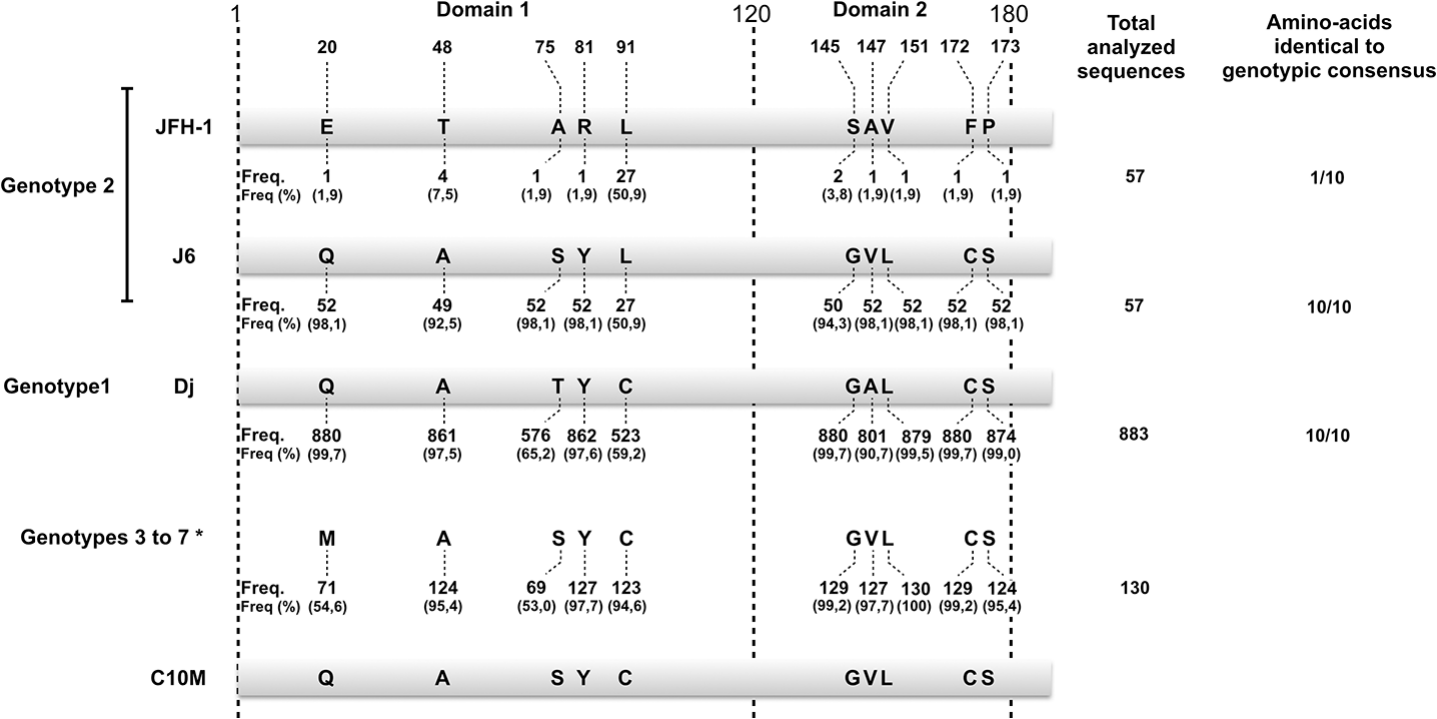
Analysis of the 1066 HCV core protein sequences extracted from the HCV Los Alamos database. Each line represents the core protein sequence of a strain (highlighted in gray) or a core protein consensus sequence (no highlighting), with a representation of amino-acid polymorphism at the 10 positions identified. Core protein domains and amino-acids positions are shown at the top. The real residue frequency (**Freq.**) or the frequency calculated as a percentage (**Freq (%)**) of all the sequences analyzed (first right column) is shown below each amino-acid. *This line corresponds to a consensus sequence from 130 core protein sequences, including 13 genotype 3 sequences, 41 genotype 4 sequences, 5 genotypes 5 sequences, 70 genotype 6 sequences and 1 genotype 7 sequence.

In total, 1224 HCV genome sequences, for genotypes 1 to 7, were first imported into an in-house database. We discarded incomplete or aberrant sequences, and finally retained 1066 complete genomes, corresponding to 883 genotype 1 sequences, 57 genotype 2 sequences, and 130 sequences of viruses of other genotypes. The core sequences were translated *in silico* and the protein sequences obtained were compared with three reference sequences: JFH-1 [2], J6CF [38] and Dj, a genotype 1 core protein that has been shown to self-assemble into HCV-like particles (HCV-LPs) at ER membranes [37]. The Dj and J6 sequences closely resembled the consensus genotype 1 and 2 sequences, respectively, whereas the JFH-1 sequence contained 10 residues (positions 20, 48, 75, 81, 91, 145, 147, 151, 172 and 173) very different from those in the genotype 2 consensus sequence and with a low to extremely low conservation frequency (Fig 1). By contrast, the amino-acids at these positions in the J6 and Dj sequences were highly conserved (between 50.1% and 99.7% identity to the genotypes 2 and 1 sequences, respectively). This analysis thus revealed that the JFH-1 core protein sequence displayed unusual features, including a combination of seven unique residues (not found in any other genotype 2 sequences) and two uncommon residues (Thr and Ala in positions 48 and 75, with respective frequencies of 4/57 and 2/57) (Fig 1). Further analysis of the sequences of viruses of other genotypes showed that the amino-acid in position 20 was highly variable but that those in the other nine positions were identical to those in the genotype 1 or genotype 2 consensus sequences (Fig 1). The 10 unusual residues in the JFH-1 sequence were equally distributed between domains D1 and D2, but absent from domain D3. Previous studies have shown that the residue in position 20 is located in the RNA-binding domain [42], the residues in positions 75, 81 and 91 are part of the putative homodimerization domain [12,43], and the last five residues—145, 147, 151, 172 and 173—are present in the D2 domain, which is structured into two α-helices, resulting in association with the membrane and LDs [14]. Following this analysis, we developed a JFH-1-derived core mutant (C10M in Fig 1), in which these 10 amino-acids were replaced by the most highly conserved residues of the genotype 1 and/or 2 consensus sequences.

### Analyses of the subcellular distribution of HCV core proteins and their ability to form HCV-like particles (HCV-LPs)

**Fig 2:**
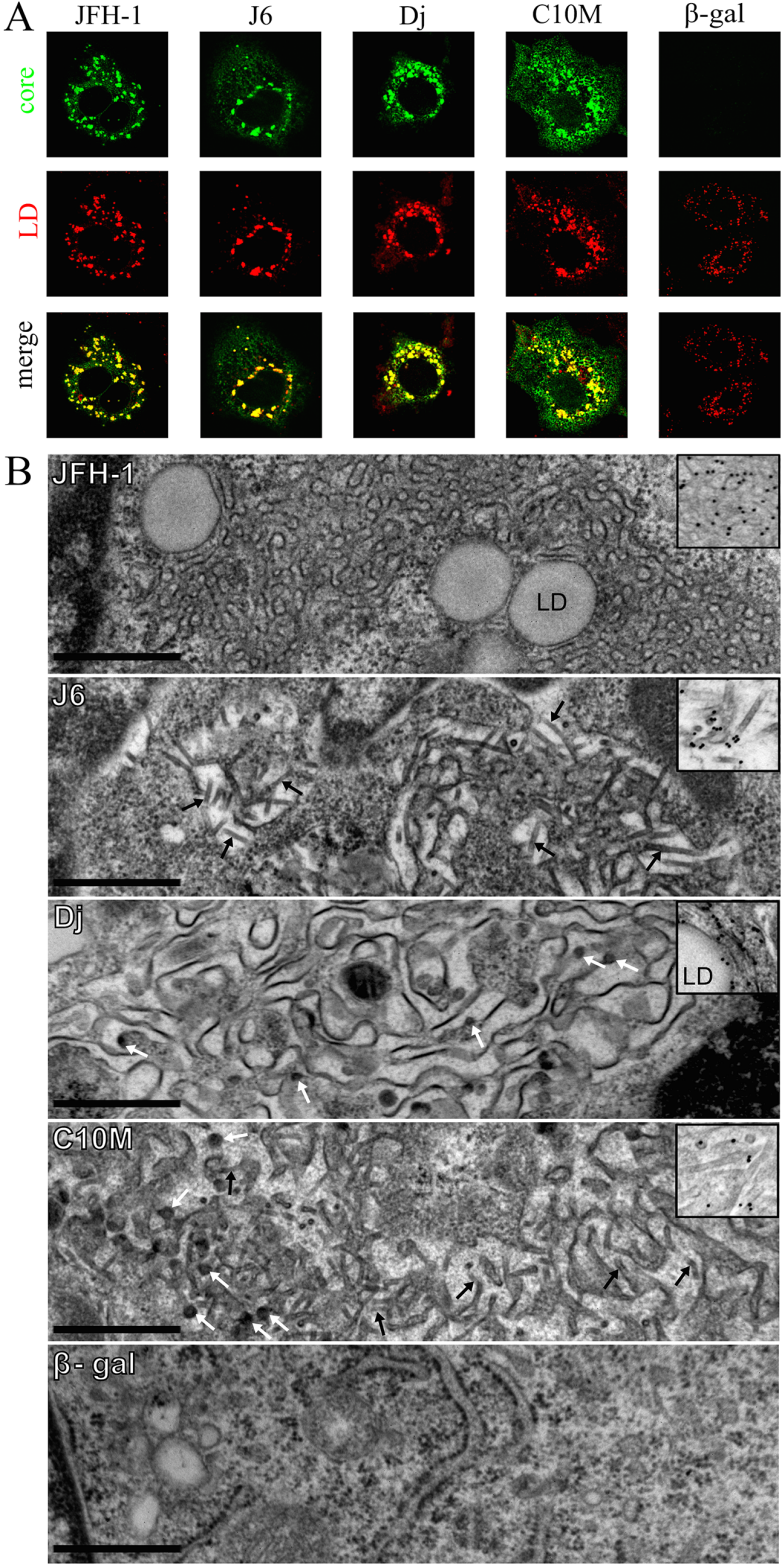
Subcellular distribution of the various HCV core proteins expressed with SFV vectors and analysis of their ability to self-assemble into HCV-like particles. **(A)** confocal microscopy of transfected FLC4 cells stained for core protein (green) and lipid droplets (red). The production of β-galactosidase (β-gal) from an SFV vector was used as a control. All images were recorded using an oil-immersion 63X of magnitude lens, and are provided as raw data (same acquisition size and conditions). **(B)** Electron micrographs of BHK-21 cells producing Dj, JFH-1, C10M or J6 HCV core. The production of any of these core proteins induced the circumvolution of ER membranes (*). The budding of HCV-LPs at these convoluted ER membranes was observed with the Dj core protein (white arrows, Dj panel), whereas oligomerization of the J6 core protein led to the formation of tubular structures (black arrow, J6 panel). No HCV-LPs or tubular structures were observed with the JFH-1 core protein. The production of the C10M core protein led to the formation of both HCV-LPs (white arrows) and tubular structures (black arrows) (C10M panel). The high-magnification insets in the four panels correspond to core protein detection by immunogold labeling, with the C7–50 anti-HCV core monoclonal antibody. Bars: 5 μm. LD: lipid droplet.

These analyses were carried out with full-length core protein sequences (191 amino-acids) expressed in two different cell lines. The FLC4 cell line [44] was used to evaluate the colocalization of core protein with LDs, whereas EM was conducted on BHK-21 cells. Expression from SFV vectors was less efficient in FLC4 cells than in BHK-21 cells, making it possible to carry out a more precise analysis of the subcellular distribution of the protein [7]. By contrast, the high level of expression obtained with SFV vectors in BHK-21 cells has been shown to be essential for the visualization of HCV-LP assembly [9,37]. Nile red labeling in control FLC4 cells (Fig 2A) transfected with β-Gal RNA showed that LDs were evenly distributed throughout the cytoplasm (Fig 2A, column 5). By contrast, transfection with the Dj core construct led to a clustering of large LDs in the perinuclear area, strongly colocalizing with the Dj core protein (Fig 2A, column 3), as previously reported [7,27]. As expected, a similar pattern was observed for expression of the JFH-1 and J6 core proteins (Fig 2A columns 1 and 2). The C10M core protein was strongly colocalized with LDs, indicating that the amino-acids substitutions in this protein (particularly those in domain 2) had no detectable effect on its properties (Fig 2A, column 4). All the core proteins had several features in common, with a typical subcellular distribution, on ER membranes and the surface of LDs, as previously described [6,7,31].

By contrast, EM analysis revealed very different ultrastructural patterns associated with the production of the various core proteins (Fig 2B). In accordance with previous findings [7], BHK-21 cells producing the Dj core protein displayed convoluted ER membranes associated with the budding of numerous HCV-LPs (Fig 2B, Dj panel). These ultrastructural features were never observed in cells producing the irrelevant β-Gal protein (Fig 2B, β-Gal panel). In accordance with previous findings [7], immunogold labeling resulted in detection of the Dj core protein on the ER membranes, HCV-LPs, and the surface of the LDs (Fig 2B, inset of the Dj panel). However, contrasting results were obtained following production of the JFH-1, J6 and C10M core proteins. All these core proteins were unambiguously detected on the convoluted ER membranes and around LDs in immunogold labeling experiments (see, for example, the inset of the JFH-1 panel, Fig 2B), but they differed considerably in terms of their ability to initiate and drive the formation of HCV-LPs. Despite the observation of multiple ultrathin sections from at least three independent experiments, no HCV-LP formation was detected in cells producing the JFH-1 core protein. By contrast, cells producing the J6 core protein displayed many tubular structures in the cytoplasm, probably corresponding to alternative polymerization forms of this core protein, as confirmed by the specific immunogold labeling of these structures (Fig 2B, inset in the J6 panel). The C10M core protein had a profile intermediate between those of the J6 and Dj core proteins. Cells producing the C10M core protein displayed budding of spherical HCV-LPs similar to those observed in cells producing the Dj core protein, together with the tubular structures identified in cells producing the J6 core protein (Fig 2B, C10M panel). The presence of the core protein in these tubular structures was again confirmed by immunogold labeling (Fig 2B, inset of the C10M panel). These results suggest that core protein multimerization, leading to HCV-LP formation, may be highly dependent on the sequence of the core protein, despite the ability of all the core proteins tested to insert into the ER membrane and to associate with LDs.

### The C10M core protein increases infectious virus production and secretion

We investigated the impact of the 10 non-conserved residues in the HCVcc system, by inserting the coding sequence of the C10M core protein into the JFH-1 genome, in place of the wild-type JFH-1 core protein sequence, to yield the C10M-JFH-1 construct. This construct was transcribed *in vitro* and used to transfect Huh7.5 cells. The transfected cells were then compared with cells transfected with the wild-type JFH-1 strain (wt-JFH-1) and the FL-J6/JFH chimera (carrying the core to NS2 sequence of the J6 isolate in the backbone of the JFH-1 strain) [45]. A chimera based on the JFH-1 backbone was also constructed with the genotype 1 Dj core protein sequence, but no virus propagation was observed when Huh7.5 cells were transfected with this construct, which was, therefore, rapidly abandoned.

**Fig 3:**
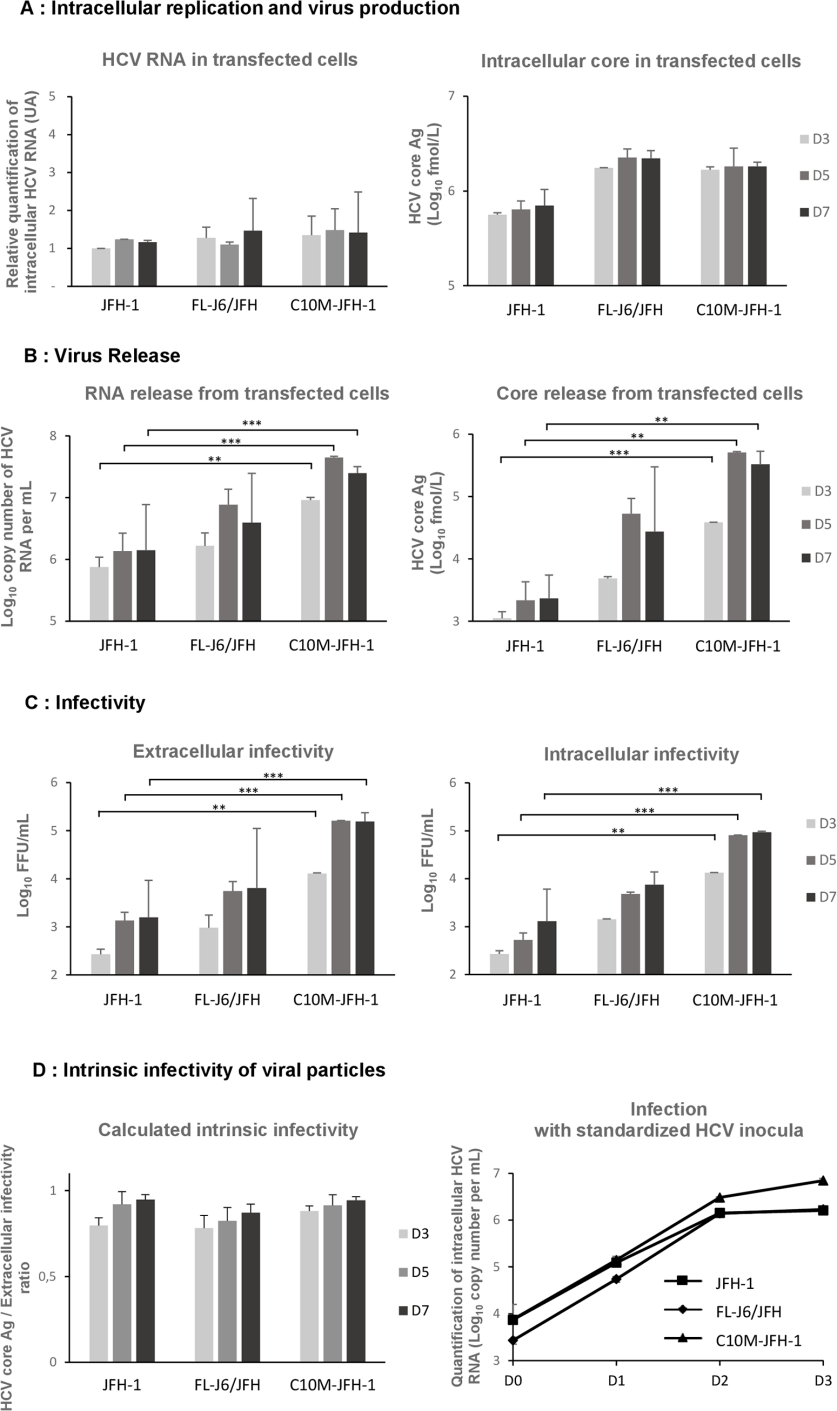
Comparative analysis at days 3, 5 and 7 of cells replicating the wt-JFH-1, FL-J6/JFH and C10M-JFH-1 RNAs. **(A)** Intracellular replication and virus production. Left panel: Replication of HCV RNAs in transfected Huh7.5 cells, as determined by RT-qPCR. Values are expressed relative to wt-JFH-1 at day 3 (D3), for which the value is fixed at 1. Right panel: Virus production in transfected Huh7.5 cells, as determined by intracellular core protein determinations with the Abbot-Architect HCV Ag. Values are normalized for intracellular core levels and RNA detection with the number of infected cells, to exclude variations caused by reinfection. **(B)** Virus release. Left panel: HCV RNA in the supernatants of transfected cells, as determined with the Abbott Realtime HCV assay. Right panel: Core protein quantification in the supernatant of transfected cells, with the Abbot-Architect HCV Ag. **(C)** Determination of the extracellular (left panel) and intracellular (right panel) infectivity of the viruses produced by transfected cells. For extracellular infectivity assays, naïve Huh7.5 cells were inoculated with supernatants obtained from cells harvested at 3, 5 and 7 days post-transfection. Intracellular infectivity was measured by assessing the infection levels of cleared cell lysates obtained following the hypotonic shock of transfected cells, on days 3, 5, and 7. The numbers of FFUs were determined after 3 days of cell culture. The mean values ± SD of three independent experiments are shown. **(D)** Analysis of the intrinsic infectivity of infectious particles produced by cells replicating the wt-JFH-1, FL-J6/JFH and C10M-JFH-1 RNAs. Left panel: Calculated intrinsic infectivity deduced from the [Core released from transfected cells] / [Extracellular infectivity] ratio from panels **B** and **C**. Right panel: After standardized infections of Huh7.5 cells with supernatants normalized on the basis of HCV RNA titration (Abbott Realtime HCV assay), intracellular HCV RNAs were quantified with the Abbott Realtime HCV assay. Mean values for one experiment performed in triplicate and means ± SD are shown. Calculated p values (Student’s t test) in **B** and **C** were presented as follows: **, p value 0.01; ***, p value 0.001.

We compared the properties of the C10M-JFH-1 construct with those of the wt-JFH-1 strain and the FL-J6/JFH chimera, by monitoring viral replication through the quantification of intracellular HCV RNA by RT-qPCR at various time points after RNA-normalized transfection (day 3, 5 and 7) (Fig 3). As previously reported [46], intracellular viral RNA levels were similar for the wt-JFH-1 and the FL-J6/JFH chimera (Fig 3A), reflecting similar replication efficiencies. As expected, similar levels of intracellular C10M-JFH-1 RNA were also observed, indicating that the replacement of the JFH-1 core protein with the C10M core protein had no significant impact on viral RNA replication. Intracellular core protein levels were similar for FL-J6/JFH and C10M-JFH-1, and slightly higher than those observed for wt-JFH-1 (Fig 3A). This may reflect slightly lower levels of JFH-1 core protein detection in this immunoassay, due to its specific sequence, but further investigations are required to determine whether this is indeed the case. Nevertheless, this first set of results indicates that these three genomes replicate with similar overall efficiencies.

We then investigated the virus assembly and release induced by these constructs. Transfection with the FL-J6/JFH chimera and the C10M-JFH-1 construct resulted in the release of larger amounts of HCV RNA into the filtered supernatant than for the wt-JFH-1 strain (Fig 3B). The amounts of HCV RNA detected were largest for the C10M-JFH-1 construct on day 5, the amounts of HCV RNA released from cells transfected with the FL-J6/JFH chimera and wt-JFH-1 being lower by factors of about 10 and 50, respectively. In parallel, similar results were obtained for the quantification of released core protein (Fig 3B), with levels of core protein release about one and two orders of magnitude lower for the FL-J6/JFH chimera and the wt-JFH-1 strain, respectively, than for the C10M-JFH-1 RNA. For confirmation of these results and to exclude the possibility of nonspecific release of viral RNA or core protein into the supernatants, we infected naïve Huh7.5 cells with the supernatant of these transfected cells collected on day 3, 5 and 7 and determined the infectivity titers of the viruses released on day 3. Infectivity titers were highest for the C10M-JFH-1 virus, with the FL-J6/JFH and wt-JFH-1 viruses having 50-fold and 100-fold lower infectivity titers, respectively (Fig 3C). As the differences in these infectivity titers were similar to those observed for virus titers, we hypothesized that the viruses released by transfected cells were of similar infectivity. We tested the hypothesis of better assembly for the viruses produced by the C10M-JFH-1 and FL-J6/JFH constructs, by assessing the intracellular infectivity of the cell lysate supernatants obtained by hypotonic shock and brief centrifugation to remove cell debris. Similar patterns were observed (Fig 3C), with differences in FFU of 1.5 orders of magnitude between C10M-JFH-1 and FL-J6/JFH and of 2.5 orders of magnitude between C10M-JFH-1 and wt-JFH-1. Moreover, intrinsic infectivity estimated from the [Core released from transfected cells] / [Extracellular infectivity] ratio was similar for the three types of viral particles (Fig 3D), suggesting that the increased infection of cells with the C10M-JFH-1 was a consequence of a more efficient viral assembly. To reinforce this hypothesis, we performed standardized infections with equivalent inocula of the different viruses (normalized by determination of HCV RNA titers). Intracellular HCV RNA quantification was carried out on the day after infection, to minimize the effect of re-infection. We found that the three HCV RNAs tested had very similar replication levels until day 2 (Fig 3D). This confirmed that the virions secreted by cells transfected with these three constructs had similar infectivity. These data are consistent with the J6 and C10M core proteins being more competent for viral assembly, rather than with a greater specific infectivity of viral particles. Our results also highlight the potentially lower efficiency of viral morphogenesis and secretion for the JFH-1 core protein (statistically significant, see Fig 3B and 3C ), consistent with a negative impact of the 10 unusual residues identified in its protein sequence.

### Viruses produced from the C10M-JFH-1 construct propagate more efficiently in Huh7.5 cells

**Fig 4:**
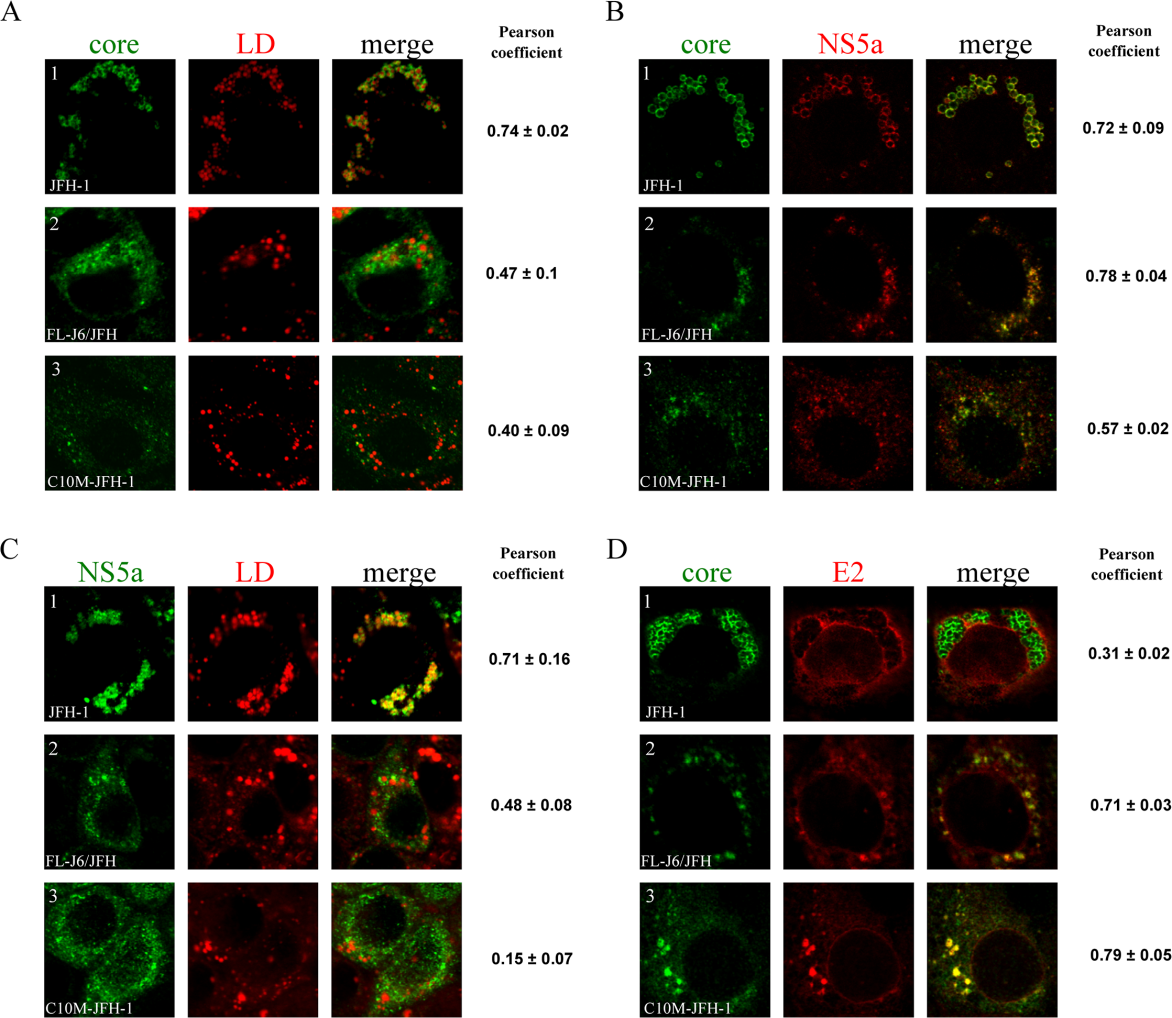
Subcellular localization of HCV viral proteins and lipid droplets by confocal microscopy. Huh7.5 cells were transfected with *in vitro*-transcribed HCV RNAs and were grown on coverslips. Cells were fixed 5 days post-transfection and analyzed for the immunofluorescence of **(A)** core protein and LDs; **(B)** core and NS5A proteins; **(C)** NS5A and LDs and **(D)** core and envelope E2 proteins. Immunofluorescence analyses were performed with the C7.50 anti-core monoclonal antibody, the AR3A anti-E2 monoclonal antibody, and anti-NS5A polyclonal antibodies. LDs were stained with Nile red, as described in the materials and methods. The value ± SD right to the image series indicates the extent of colocalization from confocal images stacks of 5 independent cells, measured as the Pearson coefficient in the cell volume dataset.

We then transfected Huh7.5 cells in normalized conditions (same amount of input viral RNA), and determined the percentage of infected cells at three time points, by immunofluorescence staining for core protein. Highly reproducible results were obtained in several independent experiments showing that, on day 5, more than 90% of the cells replicating the C10M-JFH-1 construct expressed core protein, this percentage reaching almost 100% by day 7 (S1 Fig). By contrast, an analysis of cells replicating the wt-JFH-1 strain and, to a lesser extent, the FL-J6/JFH chimera, revealed the percentage of infected cells to be lower at all time points tested. The percentage of wt-JFH-1-replicating cells is known to increase after long-term cell culture, due to the occurrence of adaptive mutations in the viral genome sequence. Our results for the C10M-JFH-1 RNA therefore suggest that this construct may produce a virus with a higher global fitness level. LDs are known to be required for core trafficking and subsequent viral assembly [26]. We therefore performed confocal microscopy analyses, to determine the subcellular distribution of the viral proteins in the context of the cells producing these different viruses. Major differences in the association of core protein with LDs were observed between cells producing different viruses (Fig 4A). Strong colocalization of the core protein with LDs was detected in cells replicating the wt-JFH-1 genome, but this interaction was much weaker in cells replicating the FL-J6/JFH genome and was almost undetectable in cells replicating the C10M-JFH-1 genome. Surprisingly, we noted that the distribution of the C10M core was particularly homogenous throughout the cytoplasm and was correlated with a lower fluorescence signal level in comparison to those obtained with the wt-JFH1 and to a lesser extent with the FL-J6/JFH replicating cells (as seen for example in Fig 4A). Since the antibody used for immunofluorescence has the same apparent efficiency for detection of the three core proteins (see Fig 2A), the diffuse distribution of the C10M core and its lower detection suggests that this protein remains mostly associated with ER membranes. Determination of Pearson coefficient showed a lower colocalization of the J6 core protein with LDs in comparison to value obtained with JFH1 core protein (Fig 4A). For the interaction of C10M core protein with LDs, the Pearson coefficient was lower than that obtained with JFH1 replicating cell, but was to our surprise very similar to that observed with the J6 core protein. Nevertheless, we could not exclude that these results might be slightly biased, as the Nile red used to stain LDs has also a significant affinity for membrane lipids bilayers, in which a substantial part of core protein remains anchored. For a better reliable quantification of this phenomenon, total LDs from 20 infected cells per construct were counted and the percentage of LDs surrounded by the core protein was determined. Cells replicating the C10M-JFH-1 genome had the lowest percentage of LDs surrounded by core protein, at 4.5% ± 2.0, whereas this percentage was 21.5% ± 9.8 and 91.5% ± 7.2 in cells replicating the FL-J6/JFH and wt-JFH-1 genomes, respectively. Together, our results demonstrate an inverse correlation between extracellular viral titers and HCV core protein association with LDs.

The NS5A protein is also detected at the surface of LDs and is thought to deliver viral RNA to the core protein [29,47]. We therefore analyzed the subcellular distribution of this protein. As expected, cells replicating the wt-JFH-1 genome displayed a strong colocalization of fluorescence signals for the NS5A and core proteins at the surface of LDs (Fig 4B and 4C). Colocalization of the core and NS5A proteins was also observed for the FL-J6/JFH construct, but this colocalization was clearly less associated with the LD surface (Fig 4B, lane 2). Finally, in cells replicating C10M-JFH-1, a partial and punctate colocalization of these two proteins suggested that most of the NS5A protein remained associated with ER membranes (Fig 4B, lane 3). This was subsequently confirmed by the absence of NS5A at the LD surface observed in cells replicating the C10M-JFH-1 genome and, to a lesser extent, in cells replicating the FL-J6/JFH chimera, by contrast to cells replicating wt-JFH-1 genome (Fig 4C). These results were confirmed with the observation of a continuous diminution of Pearson coefficients between JFH1, FL-J6-JFH and C10M-JFH1 replicating cells (Fig 4C). Despite we show that cellular distributions of NS5A and core proteins highly vary with respect to the viral RNA used to transfect cells, Pearson coefficients presented in Fig 4B showed as expected that core protein remain substantially colocalized with NS5A, regardless of the core sequence.

As the HCV particle acquires its E1 and E2 envelope proteins during the budding phase, we next performed confocal analyses to investigate the cell localization of core in comparison with that from the E2 envelope protein, known to be anchored at the ER membrane [25]. Sharp differences between the constructs were again observed (Fig 4D). In cells replicating the wt-JFH-1 genome, weak colocalization of the core and E2 proteins was detected in the form of small patches, probably corresponding to areas of intimate contact between ER membranes and LDs (Fig 4D, lane 1). By contrast, in cells replicating the FL-J6/JFH chimera and, particularly in cells replicating the C10M-JFH-1 genome, the two proteins were found to be colocalized in multiple intracellular subdomains, indicating that most of the core protein remains attached to ER membranes (Fig 4D, lanes 2 and 3, respectively). Measurement of colocalization of core with E2 proteins by determination of Pearson Coefficients showed a graduated increase of their values between JFH1, FL-J6-JFH and C10M-JFH1, thus confirming our observations. Together, these results suggest that the subcellular distribution of the NS5A protein is dependent on that of the core protein and may explain the reticular pattern of NS5A in the case of the C10M-JFH-1. Moreover, an absence of core protein at the LD surface was associated with higher levels of virus secretion for the C10M-JFH-1 construct than for the wt-JFH-1 construct, suggesting that the 10 amino-acid substitutions introduced into the C10M protein profoundly modified the intracellular trafficking of this protein, increasing virus assembly and secretion.

### The ten residues substituted in the C10M core act in a cooperative manner

**Fig 5:**
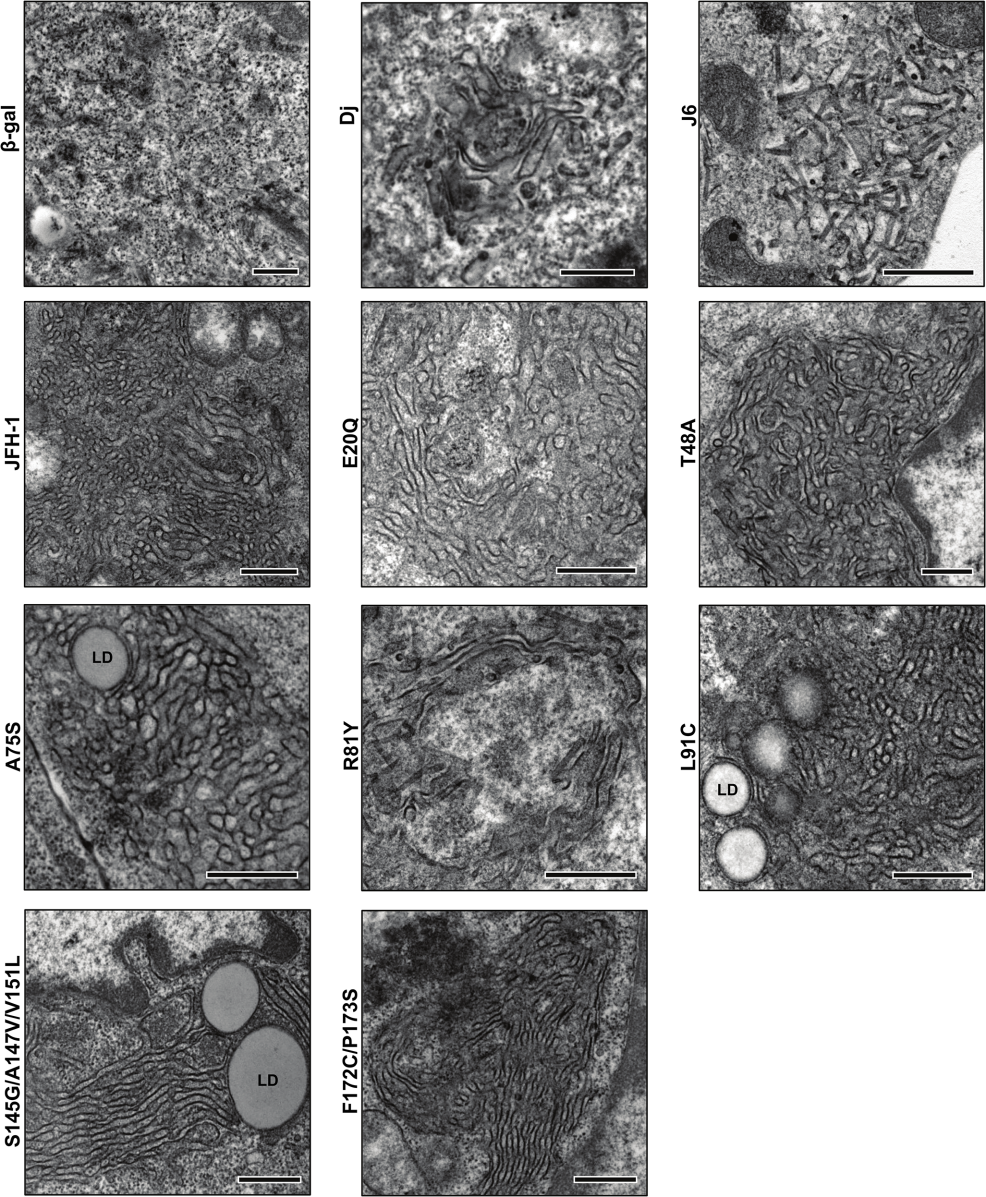
Electron micrographs of HCV core mutants expressed using SFV vectors and analysis of their ability to self-assemble into HCV-like particles. The production of all core proteins induced circonvoluted ER membranes, while no ultra-structural change of ER was identified in cells expressing the β-galactosidase, as previously described. The budding of HCV-LPs at these convoluted ER membrane was observed with the Dj core protein, while the oligomerization of the J6 core protein led to the formation of tubular structures. HCV-LPs or tubular structures were never observed with the JFH-1 core protein, as for all the core single, dual or triple mutants. Bars: 0,5 μm. LD: lipid droplet.

In order to study individual impact of residues substituted in the C10M core protein, we elaborated different mutants of the JFH-1 core carrying a single or several residues mutation. Five of these mutants included one amino-acid substitution (E20Q, T48A, A75S, R81Y and L91C), while two mutants consisted into the substitution of two or three amino-acids (S145G/A147V/V151L and F172C/P173S), as the residues located in domain 2 were previously shown to act cooperatively [31,48]. All the mutants were expressed using SFV vectors and analyzed in their propensity to traffic towards LDs as well as their ability to form HCV-LPs. Nile red labeling in transfected FLC4 cells showed that all these mutants induced a clustering of LDs in the perinuclear area, strongly colocalizing with the various core proteins, as previously shown for the Dj or the C10M core proteins (S2 Fig). EM analysis presented in Fig 5 showed that all these core mutants induced characteristic ER circonvoluted membranes. However, none of these single, dual or triple residues mutants were able to initiate HCV-LPs budding. In particular, the combination of residues previously shown to increase HCV titers in HCVcc propagation system (represented by our S145G/A147V/V151L and F172C/P173S mutants) [31,48], or the L91C mutation which is specific of the C10M in comparison to the J6 core sequence, were not able to restore the HCV LPs formation when inserted in the JFH-1 core sequence. This suggests that most if not all of the 10 conserved residues contribute in a dependent manner to the viral assembly process.

## Discussion

Much has been learnt about the lifecycle of HCV in recent years, but many aspects relating to virus assembly and secretion remain unclear. The three structural proteins (core, E1 and E2) have been implicated in these processes, but recent reports have shown that almost all viral proteins may be involved in virus assembly, in addition to several host cell proteins such as DGAT-1, rab18, TIP47 and several proteins involved in VLDL synthesis, [20,49–51]. In this study, we focused on the JFH-1 core protein, as this protein has repeatedly been shown to be limiting for virus production in the HCVcc system [31,34]. We compared the sequence of the JFH-1 core protein with those of other core proteins, determined by *in silico* translation from all the complete HCV genome sequences available from databases, presumably derived from functional circulating viruses isolated from infected patients. This approach is original, as most previous studies have focused on comparison of the JFH-1 core protein sequence with selected canonical sequences, such as the J6 core protein sequence, which may not reflect sequence diversity. We identified 10 unusual residues in the JFH-1 core protein rendering this sequence unique, not only to genotype 2, but to HCV in general, regardless of the genotype considered (Fig 1). Several of these 10 residues, including the alanine and valine residues identified in positions 147 and 151 respectively [31], or the phenylalanine and serine residues in positions 172–173 [48], have been studied before. Interestingly, it has already been suggested that these four residues are responsible for the partial defect of the JFH-1 core protein in viral assembly. Nevertheless, as most of these 10 residues remain to be investigated and considering that most, if not all of these residues have coordinated and additive effects on viral assembly, we studied the properties of a C10M core protein in which these 10 residues were replaced with the most conserved residues (Fig 1). We thus compared the fitness of a C10M-JFH-1 strain encoding this mutated C10M core protein with the parental JFH-1 strain.

Using our model of HCV-LP assembly based on HCV core protein production with an SFV vector [9,27,52], we first evaluated the ability of this C10M protein to self-assemble into HCV-LPs, comparing our results with those for the Dj, J6 and parental JFH-1 core proteins. We found that the JFH-1 core protein could not form HCV-LPs, but that the 10 substitutions introduced into the C10M mutant were sufficient to restore HCV-LP formation. Surprisingly, although production of the Dj core protein led to the formation of numerous HCV-LPs budding at the ER membranes, as previously reported [9,27,52], production of the J6 core protein was associated with the formation of many core-related tubular structures. This observation probably reflected a combination of several factors, such as overproduction of the protein, the absence of the other viral components, or higher degrees of multimerization, as previously described for the HIV-1 capsid protein [53,54] or the rotavirus VP6 protein [55]. The formation of tubular structures following production of the J6 core protein and, to a lesser extent, the C10M core protein, for which tubular structures were observed together with the HCV-LPs, contrasting with the formation of HCV-LPs alone observed with the Dj core protein, remains puzzling and requires further investigation. Nevertheless, our results indicate that the native JFH-1 core protein is defective for HCV-LP formation, highlighting the negative impact of its 10 non-conserved residues on viral assembly. This defect was readily overcome by engineering selected substitutions of these residues. Delineating the individual impact of these substitutions in the budding process failed to identify one or several amino-acids directly involved in viral morphogenesis. Although point mutations were tested for their impact in HCV-LPs assembly in the SFV system rather than in the HCVcc model, these results suggests that most of these substitutions if not all have synergic and additive effects in the C10M core protein on promoting the viral assembly and may explain its high viral assembly/secretion properties in the HCVcc model. Moreover, although all the core proteins tested were trafficked to LDs with similar efficiencies, we found that HCV-LP assembly was always unambiguously detected at ER membranes, but never at the surface of the LDs, suggesting that these ER membranes were probably the site of core multimerization and nucleocapsid assembly.

The beneficial effects of these 10 amino-acids substitutions on virus assembly and release were confirmed by analysis of the C10M mutant core protein in the context of the HCVcc system. Indeed, a strain in which a sequence encoding the C10M mutant core protein was inserted into the backbone of the JFH-1 genome displayed higher levels of intracellular and extracellular infectivity than the wt-JFH-1 strain. The higher viral titers obtained with the C10M-JFH-1 genome than with the FL-J6/JFH chimera suggested that the HCVcc system could be optimized by several mutations of the core protein sequence, resulting in virus production levels even higher than were achieved with chimeric genomes. This suggests that the E1 and E2 envelope proteins, and the p7 and NS2 proteins of the wt-JFH-1 strain are as efficient as those from the J6 strain, in viral assembly. It has recently been shown that E1 and E2 from the wt-JFH-1 and J6 sequences are similar in terms of their infectivity [56]. We show here that the E1, E2, p7 and NS2 proteins from the wt-JFH-1 strain are fully functional for efficient virus assembly and secretion.

Soon after its synthesis on ER membranes, the HCV core protein accumulates on the surface of LDs in wt-JFH-1 HCVcc-infected cells [6,26,28]. It has been suggested that LDs might cause core protein to accumulate close to the ER, generating a favorable environment for the recruitment of viral RNA and assembly of the nucleocapsid [6]. Confocal imaging showed that, in the context of the HCVcc system, the C10M core protein mutant was barely detectable at the LD surface, instead being found in the ER membranes, in which it was extensively colocalized with the E2 protein (Fig 4D). This pattern was similar to that for the J6 core protein, but contrasted sharply with that for the JFH-1 core protein, which was detected mostly at the LD surface. Our results confirm that a strong association of core protein with LDs is accompanied by poor assembly into infectious particles [31,34–36]. Low levels of core protein on LDs, initially described with constructs highly efficient for viral assembly, such as the Jc1 chimera [46] or a modified JFH-1 strain encoding the J6 core protein [31], have been interpreted as demonstrating the rapid trafficking of core protein between the ER membranes and LDs, enhancing viral assembly [31]. Recent studies analyzing the HCV core proteins produced by chimeric genomes bearing sequences encoding the core to NS2 proteins from various genotypes [35] or by a genotype 3 strain adapted to cell culture [36] have shown that these proteins are weakly associated with LDs, unlike the JFH-1 core protein. These observations and the data presented here suggest that the JFH-1 core protein sequence is extremely unusual among HCV core protein sequences.

The data we obtained with the C10M-JFH-1 strain suggest that the HCV core protein may be incorporated into nascent particles immediately after its synthesis, possibly without passing via the LD surface. Alternatively, the C10M core protein may associate extremely briefly with the LD surface, thereafter being trafficked back to the ER membranes to form viral particles. This hypothesis is supported by our SFV experiments, showing that the C10M core protein assembles into HCV-LPs at ER membranes, even though it can be trafficked to LDs. The strong association of the JFH-1 protein with LDs might be not optimal for virus assembly. Current models suggest that LDs may act as platforms facilitating the local concentration of viral components for the initiation of assembly, as shown for the presence of the core and NS5A proteins on LDs [29,47]. However, in the case of our C10M-JFH-1 strain, the core and NS5A proteins were found colocalized mostly on the ER membranes rather than on the surface of LDs. As the interaction of core protein with NS5A is thought to occur early in assembly, this suggests that these early assembly events occur at ER membranes distant to the LDs. Our results also suggest that core protein is the major determinant of the subcellular distribution of the NS5A protein, because our C10M core protein mutant and, to a lesser extent, the J6 core protein, can redirect the NS5A protein from LDs to the ER membranes. These results are consistent with those of Galli *et al*. [35], who reported that the core and NS5A proteins encoded by chimeras of various genotype sequences colocalized mostly at sites other than the LD surface, contrasting with the situation observed for the JFH-1 strain.

The viruses produced by the different constructs studied had a similar intrinsic infectivity, but almost 100% of the cells replicating the C10M-JFH-1 genome were infected at day 5, indicating that this particular genome produced viruses of greater fitness. Such differences in fitness have already been reported following comparisons of propagation rates between viruses produced from wt-JFH-1 and the Jc1 chimera [34], but the C10M-JFH-1 genome may be a particularly well optimized genome for viral propagation. This could reflect better multimerization of the C10M core protein and/or better recruitment of the core protein to NS2-containing ER membranes, through a putative interaction with p7. However, this association remains to be clarified, because direct core-p7 and core-NS2 interactions, although suspected, have never been reported to date [32,34,57].

In conclusion, we have shown that the JFH-1 core protein carries 10 non-conserved residues that impede virus assembly and secretion. The replacement of these 10 residues with residues conserved at the corresponding positions in other HCV strains led to high levels of virus release. The subcellular localization of the protein to the ER membranes rather than the surface of the LDs, and a better ability to multimerize and to self-assemble into viral particles appear to be the principal properties of this modified core protein accounting for this phenotype. The C10M-JFH-1 strain generated here will be a valuable tool for further studies of HCV morphogenesis, which remains the least well understood step in the HCV lifecycle.

## Supporting Information

S1 Fig Determination of the percentage of infected cells by immunofluorescence staining of core protein 3, 5 and 7 days after transfection with a normalized amount of *in vitro*-transcribed RNAs. Mean values ± SD from three independent experiments are shown.
(TIFF)

S2 Fig Subcellular localization of the various HCV core proteins mutants expressed using SFV vectors. Transfected FLC4 cell stained for core protein (green) and lipid droplets (red). The production of β-galactosidase **(β-gal)** with an SFV vector was used as control. All images were done with an oil-immersion 63X of magnitude lens, and are provided as raw data (same acquisition size and conditions).
(TIFF)
