# Supplementary figures and images for: The Replacement of 10 Non-Conserved Residues in the Core Protein of JFH-1 Hepatitis C Virus Improves Its Assembly and Secretion
Source: PLoS One. 2015 Sep 4;10(9):e0137182. doi: 10.1371/journal.pone.0137182 (PMC4560444; doi:10.1371/journal.pone.0137182)

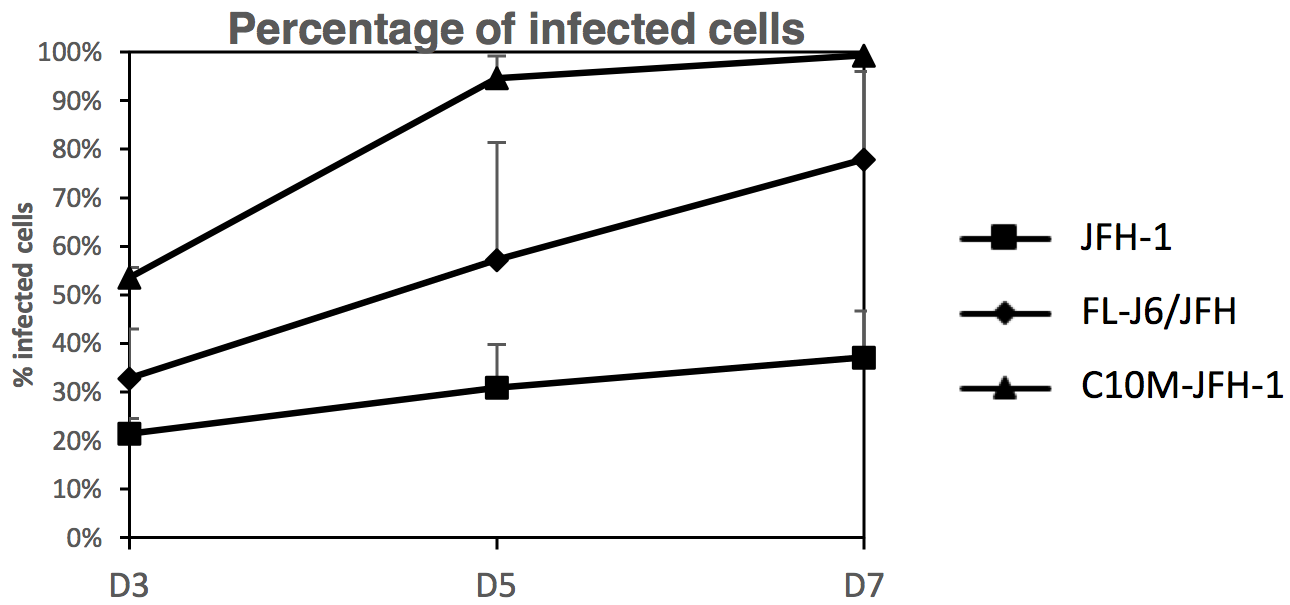

Supplement: S1 Fig — Mean values ± SD from three independent experiments are shown. (TIFF) [file pone.0137182.s001.tiff]
